# Cognitive failure, teacher’s rejection and interpersonal relationship anxiety in children with dyslexia

**DOI:** 10.12669/pjms.313.7065

**Published:** 2015

**Authors:** Aneeza Habib, Fauzia Naz

**Affiliations:** 1 Aneeza Habib, Department of Applied Psychology, Queen Marry College, Lahore, Pakistan; 2 Fauzia Naz, Ph.D Assistant Professor, Department of Applied Psychology, Queen Marry College, Lahore, Pakistan

**Keywords:** Dyslexia, Cognitive failure, Interpersonal relationship anxiety, Teachers’ rejection

## Abstract

**Objectives:**

Present research aimed to explore relationship between cognitive failure, teacher’s rejection (TR), interpersonal relationship anxiety (IRA) and Signs of Dyslexia (SD) in children with dyslexia. Another aim was to explore TR, SD and IRA as predictors of cognitive failure and final aim was to see TR, SD and cognitive failure as predictors of IRA.

**Method:**

Sample included140 students (70 girls & 70 boys) with dyslexia. Their age was ranged between 7-14 years (mean age: M=10.50, SD= 1.89). Cognitive Failure Questionnaire, Teacher’s Acceptance-Rejection Questionnaire and Interpersonal Relationship Anxiety Questionnaire were used for assessment.

**Results:**

Results revealed significant positive relationship between cognitive failure, TR, SD and IRA. TR, SD, IRA emerged as significant predictors of cognitive failure while TR, SD and cognitive failure emerged as significant predictors of IRA.

**Conclusion:**

Findings from the present research have practical implications for parents, teachers, trainers and health physicians while dealing children with dyslexia.

## INTRODUCTION

Dyslexia is a specific learning disability (LD) that has a neurobiological origin^1^ and is characterized by difficulties in reading, spelling, writing, speaking and understanding.^2^ It is an inability to process graphic symbols and appears to be related to age, social and emotional issues and deficits in cognitive abilities.^3^

Dyslexia includes substantially below reading level which significantly interferes with academic achievements.^2^ The prevalence rate of dyslexia ranges from 5.0 to 17.5 percent in children and adults.^4^

The children with dyslexia also exhibit problems in organizing spoken language, recognizing and memorizing letters and sounds and solving math problems. In addition, they have difficulties in reading and writing.^5^ These children often display behavioral problems and have low self-esteem. They have sometimes cognitive limitations as well as issues with attention span. They often get frustrated in daily routine and during academic activities at school.^6^

The causes of dyslexia may be neurobiological, experiential and instructional. In Pakistan, 10-18% children suffer from dyslexia.^7^ Due to deficient automatic information-handling processes,^8^ they encounter problems in cognition and suffer from distorted memory,^9^ fail in performing routine learning activities.^10^ As a result, their working memory gets impaired.^11^

Owing to poor concentration in class-room, they may face rejecting behavior from teachers.^12^ Rejection from teachers has been linked to emotional disturbances i.e., anxiety symptoms resulting into negative educational outcomes.^13^ So children, who have learning difficulties in combination with cognitive/attention deficits and perceived rejection from teachers, may become vulnerable to develop anxiety symptoms, may increase their cognitive and attention deficits and resultantly enhance their learning difficulties.

### Rationale/significance of the study

Present study may give directions to parents/teachers/health physicians to change their communication styles while handling children with dyslexia so that these children increase confidence level, overcome their problems and become useful members of the society. Further, the present research may also be helpful for trainers to develop or plan some interventions for the children with dyslexia to help them overcome their academic problems.

### Hypotheses

1. There is likely to be positive relationship between cognitive failure, TR and IRA in children with dyslexia.
2. TR, SD and IRA will be the predictors of cognitive failure while conversely, CF, SD and TR will be predictors of IRA in children with dyslexia.

## METHODS

### Research Design and Sample

Cross-sectional research design was used to recruit sample. Sample comprised of 140 students (70 girls & 70 boys) of grade 4-8 from different high schools. Their age range was from 7 to 14 years (Mean= 10.70, SD=1.91 for girls; Mean 10.92, SD=1.87 for boys). The students were recruited after initial screening interview using CWSDC^14^ with the help of class-teachers. Those students were excluded who had any physical disability or psychological problems.

### Measures

### Dyslexia Identification Scale (DIS)

A self-constructed DIS (seven-items) was used to identify students having symptoms of dyslexia. Indicators for DIS were drawn from the 20 students of grade 4 to 8 who had academic difficulties. The items listed were the ones that have clarity of concept, relevance to the construct, have readability/comprehensibility. The response options are ‘no’, ‘sometimes’ and ‘always’.

### Common Warning Signs of Dyslexia Checklist

Grade 4 to 8 (CWSDC^14^) CWSDC is a 38 items (α=.88) checklist used by teachers to identify children with dyslexia in different areas of learning i.e., problems in language, reading, writing, social and emotional complications attributable to dyslexia. The response options are ‘yes’, ‘sometimes and ‘never’.

### Cognitive Failure Questionnaire (CFQ^15^)

CFQ (25-items; α=.89^15^; α=.81) assesses everyday slips/errors and minor mistakes which shows cognitive failure in children with dyslexia. The response options range from ‘very often’ to ‘never’.

### Teachers’ Acceptance- Rejection Questionnaire (TARQ^16^)

TARQ is 29 items (α=0.70) scale used by children to evaluate the perceived rejection of their class teachers i.e., coldness/lack of affection, hostility/aggression, indifference/neglect and rejection. The response options range from ‘always true’ to ‘never true’.

### Interpersonal Relationship Anxiety Questionnaire (IRAQ^16^)

IRAQ is a 9-item (α = 0.87^16^; α=0.80) measure which assesses anxiety symptoms. The response options range from ‘not at all’ to ‘always true’. The highest score (36) indicates severe anxiety while interacting in social situations.

### Demographic Information

Information included gender, age, education, No. of siblings, birth order, family system and family income.

### Procedure

The researcher recruited students from grade 4-8 from different public schools with the help of teachers. The students were screened out on the basis of DIS and CWSDC.^14^ The children who scored higher than mean score on CWSDC^14^ were identified as children with dyslexia. All the assessment measures were administered in the presence of researcher. Data were analyzed and results were discussed.

## RESULTS

The relationship between cognitive failure, teachers’ rejection and interpersonal relationship anxiety and signs of dyslexia in children with dyslexia employing, Correlational analyses are shown in Table-I.

**Table-I:** Relationship between CF, TR, IRA, and SD in Children with Dyslexia (N=140).

Measures	2	3	4	5	6	7	8	9	10	11	12
1. CF	0.39**	0.30**	0.26**	0.45**	0.42**	0.62**	0.25*	0.22*	0.19*	0.22*	0.12
2. H/A		0.47**	0.37**	0.27**	0.10	0.47**	0.11	0.12	0.13	0.11	0.13
3. I/N			0.33**	0.12	0.16	0.28**	0.13	0.16	0.14	0.15	0.11
4. UR				0.20*	0.13	0.23**	0.11	0.12	0.18	0.11	0.11
5. Control					0.48**	0.34**	0.13	0.11	0.13	0.11	0.10
6. Coldness						0.27**	-0.15	0.17	0.16	0.14	0.14
7. IRA							0.21*	0.23*	0.19*	0.20*	0.10
8. LD								0.25**	0.19*	0.18*	0.10
9. RD									0.25**	0.17	0.23*
10. WD										0.11	0.21*
11. SED											0.11
12. OD											

*Note:* CF= Cognitive Failure; H/A= Hostility/Aggression; I/N= Indifference/Neglect;

UR= Undifferentiated Rejection; LD= Language Disability; RD= Reading Disability;

WD= Writing Disability; SED= Social Emotional Disability, OD= Other Disability.

* p<0.05.

** p<0.01.

Results show significant positive relationship between cognitive failure and hostility/aggression, indifference/neglect, undifferentiated rejection, control, interpersonal relationship anxiety and language disability, writing disability and socio-emotional disability in children with dyslexia. Stepwise regression analyses were employed to see TR, IRA and CWSD as predictors of cognitive failure. This result are shown in Table-II.

**Table-II:** Stepwise Regression Analyses (forward) Predicting CWSDC, IRA, TR as Predictors of Cognitive Failure.

Variables	Cognitive Failure				
	Models				
	Β	Β	Β	β	Β
IRA	0.62***	0.54***	0.50***	0.49***	0.45***
Coldness/Lack of Affection		0.27***	0.19**	0.16*	0.16*
Socio-Emotional Disability			0.80*	0.20**	0.19**
Language Disability				0.16**	0.17**
Indifferent/Neglect					0.14*
R2	0.39	0.46	0.48	0.51	0.53
∆R2	0.38	0.44	0.47	0.49	0.51
F	87.34***	57.30***	41.96***	34.81***	29.75***

* p<0.05.

** p<0.01.

*** p<0.001.

Results revealed that for cognitive failure, interpersonal relationship anxiety, coldness/lack of affection, socio-emotional disability and language disability emerged as significant predictors (F= 87.34; p<.000) accounting for 53% of the variance. In order to find the CWSD^14^, TR and cognitive failure as predictors of interpersonal relationship anxiety, stepwise regression analyses were employed. Table-III shows Stepwise Regression Analyses Predicting IRA from CWSDC, TR and CF.

**Table-III:** Stepwise Regression Analyses (forward) Predicting IRA from CWSDC, TR and CF.

Variables	Models (IRA)		
	Β	Β	Β
Cognitive Failure	0.62***	0.51***	0.45**
Hostility/Aggression		0.27***	0.33**
Socio-Emotional Disability			0.22**
R2	0.38	0.45	0.40
∆R2	0.38	0.44	0.42
F	87.34***	56.27***	35.33***

*Note:* N=140,

** p<0.01.

*** p<0.001.

Results revealed that cognitive failure, hostility/aggression, and socio-emotional disabilities emerged as significant predictors of interpersonal relationship anxiety (F= 87.34; p<000) accounting for 40% of the variance.

## DISCUSSION

Present study revealed significant positive relationship between learning disabilities, cognitive failure, interpersonal relationship anxiety and teacher’s rejection. There is evidences^9^ that children with dyslexia develop symptoms of cognitive failure and have impaired memory.^11^ These findings are also supported in a research^17^ which found disturbance in the neural system of children with dyslexia in posterior brain and positive association between reading skills and the degree of stimulation in the left occipito-temporal region which enhances learning disabilities. Children with dyslexia are likely to have problems in class-room such as rejection from teachers which further causes psychological distress and enhances their social problems in class-room and outside the class.^18^ Edwards^19^ suggests that teachers should be able to recognize the frustration that children with dyslexia feel in classrooms which results from their inability to read and write.

Results from the present research revealed significant positive relationship between interpersonal relationship anxiety and language disability, writing disability, socio-emotional disability, coldness/lack of affection, indifferent/neglect, hostility/aggression, undifferentiated rejection and control from teachers. It is likely that children who face difficulties in class-room activities like learning and comprehending classroom lessons, speaking in front of teacher and other students and answering teacher may develop feelings of symptoms of anxiety. As regards facing difficulties in learning, they also perceive rejection from teachers which plays role in developing interpersonal relationship anxiety feelings. These results are in line with the previous studies^20^ that children with dyslexia exhibit confused patterns of communication and absent-mindedness especially in class-room. They have poor concentration and fail to recall homework, fail in distinguishing left from right and finding their way in an unknown place. Because of these de-regularities, children with dyslexia may face rejection from teachers or from other students at school or in class-room. So, the milieu of combination of dyslexia symptoms and rejection from teachers may contribute to develop anxiety symptoms in children with dyslexia.^12^ These findings are also supported by the findings^7^ that learning difficulties are associated with increased comorbidity, especially depression and anxiety in children with dyslexia.

Further, the results from stepwise regression analysis revealed that interpersonal relationship anxiety, coldness/lack of affection and social-emotional disabilities, language disability, and indifferent/neglect arose as significant predictors of cognitive failure accounting for 53% of the variance. The model was also overall fit (F = 29.75; p<.000). On the contrary, when we tested model to predict interpersonal relationship anxiety from cognitive failure, teacher’s rejection and signs of dyslexia, the results revealed that cognitive failure, hostility/aggression (45% of the variance) and social-emotional disability (40% of the variance) emerged as significant predictors of interpersonal relationship anxiety. The model was overall fit (F=35.33; p<000).

These results are supported by a study^17^ in which researchers found disturbance in the regions of posterior brain which involves parieto-temporal and occipito-temporal areas which impair their cognitions gravely. They also found reading skills were positively associated with the degree of stimulation in the left occipito-temporal region which causes disturbances in reading in children with dyslexia and that is more prevalent at young age.^21^ Research^22^ also describes that learning disabilities could be the significant factor in developing psychopathologies in children e.g., anxiety, depression etc. Ryan^23^ has found links between anxiety symptoms and teachers rejection and argued that when children feel anxiety before teacher, the teacher misinterprets and tags the child as indolent and inactive in class-room in front of other students who make fun of the child which further aggravates the anxiety symptoms and develops shilly shallying behavior.

## CONCLUSION

It is concluded that cognitive failure, teachers’ rejection and interpersonal relationship anxiety symptoms contribute to intensify the existing learning difficulties in children with dyslexia. Learning difficulties in children could be lessened by addressing these issues. Teachers should be educated to behave with these children in a way that they do not feel humiliated, embarrassed and uncomfortable in class-room. Teachers can enhance resilience and confidence of these children so that they may overcome their learning problems and develop improve and suitable lifestyle to become useful citizens.
